# Supplementary material for: General Trends in the Calnexin-Dependent Expression and Pharmacological Rescue of Clinical CFTR Variants
Source: bioRxiv. 2025 Sep 11:2025.04.03.647093. Preprint. [Version 2] doi: 10.1101/2025.04.03.647093 (PMC12439906; doi:10.1101/2025.04.03.647093)
Supplement: Supplement 1 [file media-1.pdf]

Supplemental Information for:

General Trends in the Calnexin-Dependent Expression and Pharmacological Rescue of Clinical CFTR Variants

Austin Tedman,[1] John A. Olson III,[2,3] Minsoo Kim,[2,3] Catherine Foye,[4] JaNise J. Jackson,[4] Eli F. McDonald,[2] Andrew G. McKee,[5] Karen Noguera,[5] Charles P. Kuntz,[1] Jens Meiler,[2,6] Kathryn Oliver,[4] Lars Plate,[2,7] and Jonathan P. Schlebach[1]*

[1] *The James Tarpo Jr. and Margaret Tarpo Department of Chemistry, Purdue University, West Lafayette, IN, USA*
[2] *Department of Chemistry, Vanderbilt University, Nashville, TN, USA*
[3] *Program in Chemical and Physical Biology, Vanderbilt University, Nashville, TN, USA*
[4] *Department of Pediatrics, Emory University School of Medicine, Atlanta, GA, USA*
[5] *Department of Chemistry, Indiana University, Bloomington, IN, USA*
[6] *Institute for Drug Discovery, Leipzig University, Leipzig, SAC, Germany*
[7] *Department of Biological Sciences, Vanderbilt University, Nashville, TN, USA*

*Corresponding Author: jschleba (at) purdue.edu

Contents

-Figure S1
-Figure S2
-Figure S3
-Figure S4
-Figure S5
-Figure S6
-Figure S7
-Figure S8
-Figure S9
-Figure S10
-Table S1

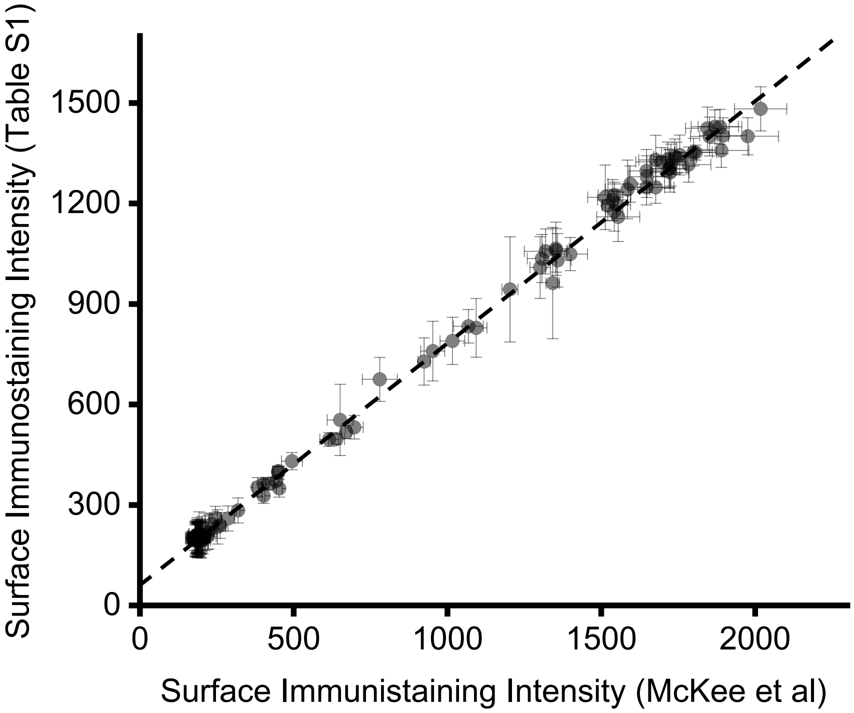

Figure S1. Comparison of Deep Mutational Scanning Measurements Derived from First and Second Generation CF Variant Libraries. A subset of surface immunostaining intensities for 129 CF variants derived from deep mutational scans using the library of 235 variants described herein are plotted against the corresponding 129 values that were previously reported from a deep mutational scan of the first generation library described in reference 5. Values represent the average of three biological replicates and error bars represent the standard deviation. A linear best fit is shown for reference (Pearson's R2 = 0.9975).

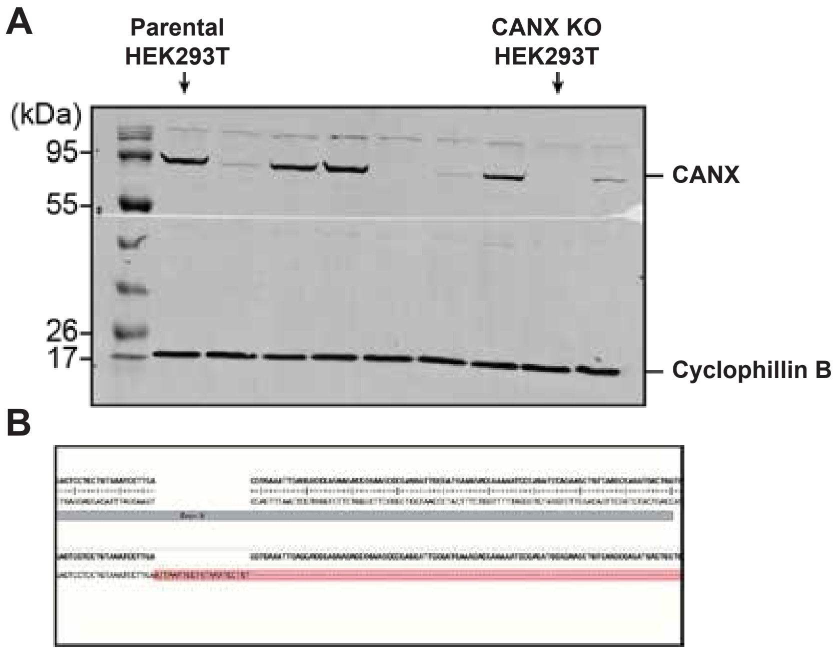

Figure S2. Validation of Cas9 Mediated CANX Knockout Cells. A) Western blot compares the levels of CANX protein expression across a series of clones isolated following transfection with a Cas9 RNP loaded with anti-*CANX* guide RNA. The lanes containing lystate from the parental cell line and the knockout line used for the studies detailed herein are indicated. An anti-cyclophillin B stain loading control is shown for reference. B) The aligment of a sanger sequencing read to the CANX gene sequence demonstrated that the chosen knockout clone indicated in panel A) contains a deletion within exon 8.

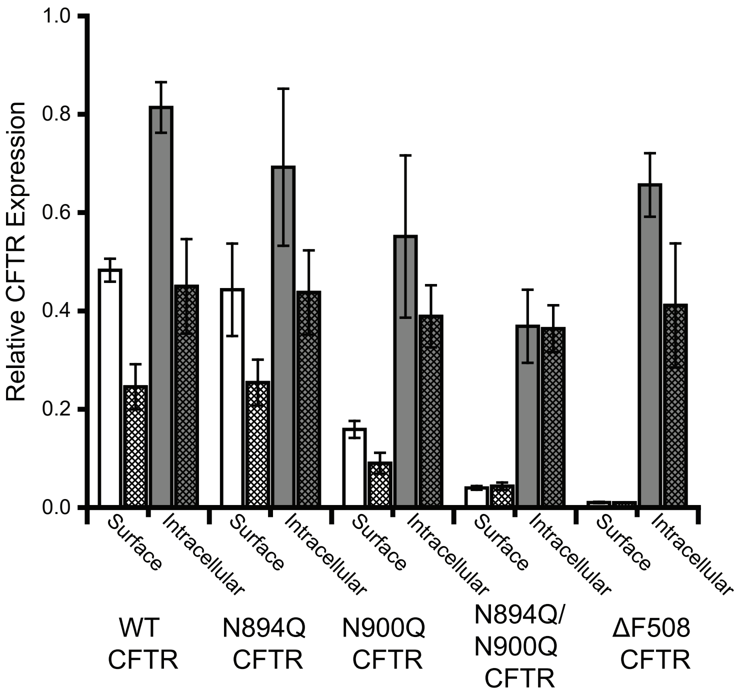

Figure S3. Surface and Internal Expression of N-Glycan Knockouts in Parental and CANX KO Backgrounds. A series of HA-tagged CFTR variants wer transiently expressed in parental HEK293T (open) and CANX KO (patterned) HEK 293T cells. Surface CFTR on intact cells was first immunostained with a Dylight-550 conjugated anti-HA antibody, then fixed and permeabilized prior to immunostaining of the intracellular CFTR with an Alexaflour-647 conjugated anti-HA antibody. Relative surface and intracellular immunostaining intensities were then measured by flow cytometry. A bar graph depicts the relative surface immunostaining intensity (white) and relative intracellular immunostaining intensity (gray) CFTR immunostaining for each CFTR variant. Values represent the average of three biological replicates and error bars represent the standard deviations. These data show that both WT and ΔF508 CFTR exhibit impaired expression in the absence of CANX. This expression defect persists for single mutants of either glycosylation site (N894 or N900). However, the CANX dependence of expression is lost for the double mutant that lacks N-linked glycosylation sites. Consistent with the findings in reference 19, these trends validate that CFTR exhibits lower expression in CANX knockout cells. Our results also suggest this expression defect depends upon its recognition of both N-linked glycans.

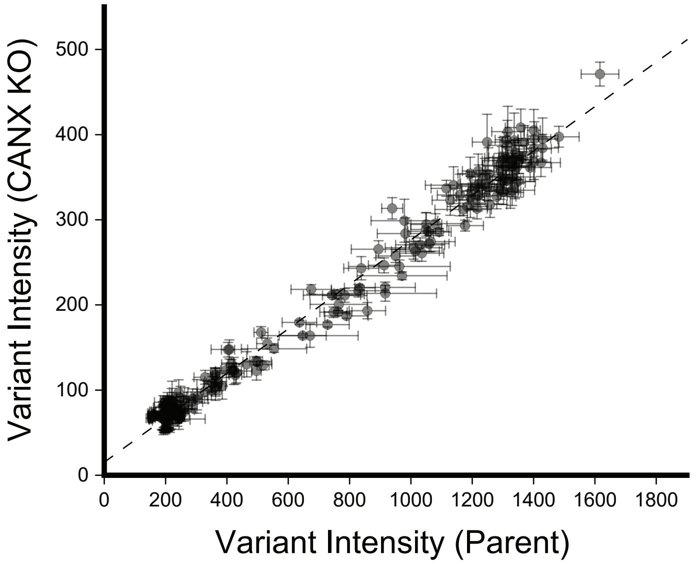

Figure S4. Plasma Membrane Expression of CF Variants in Parental HEK293T Cells and CANX Knockout HEK293T Cells. Deep mutational scanning measurements of the surface immunostaining intensities of 234 CF variants in parental HEK293T cells are plotted against the corresponding surface immunostaining intensities in CANX knockout cells. Values represent the average of three biological replicates and error bars represent the standard deviations. A line of best fit is shown for reference (*m* = 0.26).

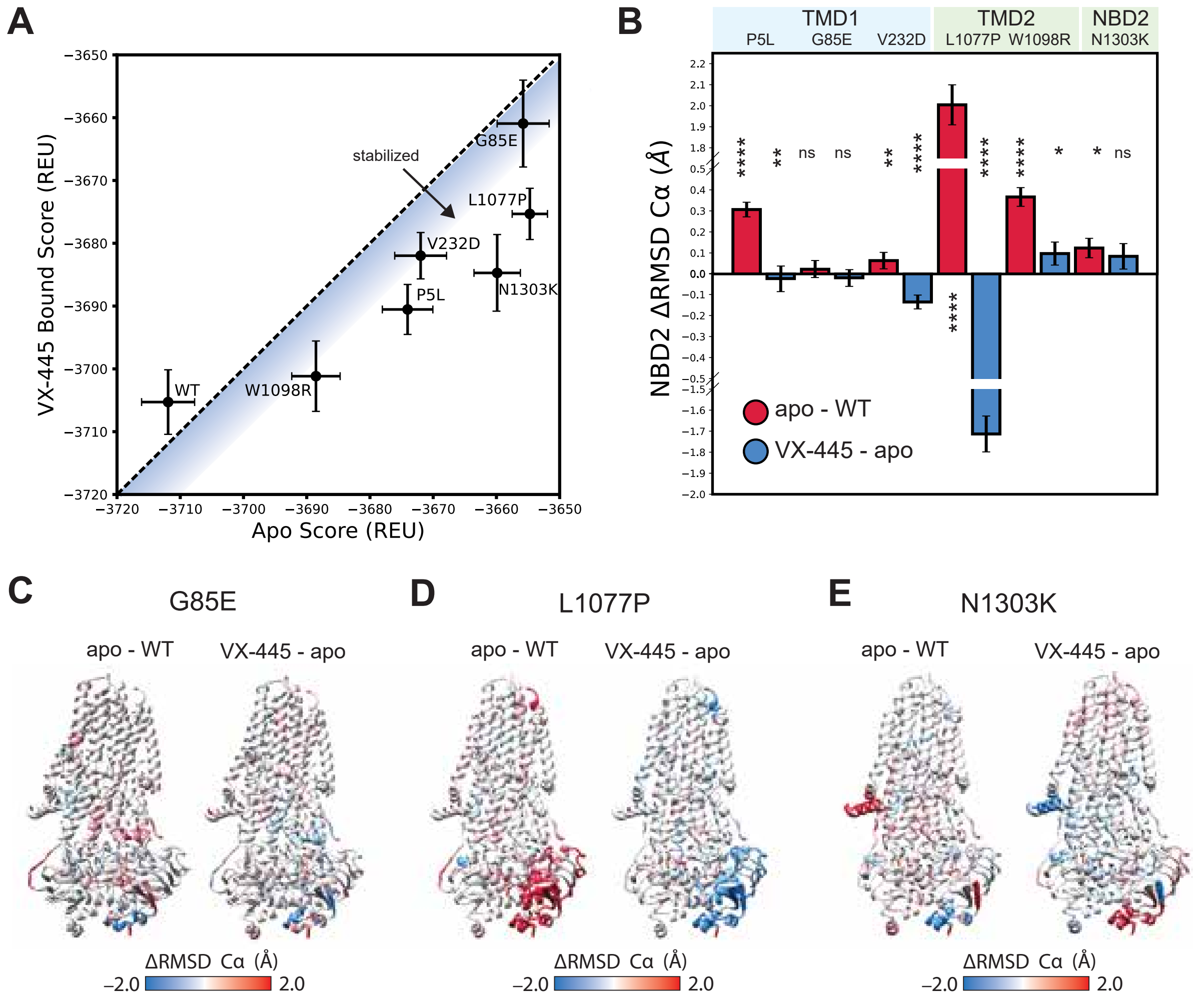

Figure S5. VX-445-Mediated Suppression of Conformational Defects in NBD2. Structural modeling was used to compare the conformational states of the apo and VX-445-bound active structures of WT CFTR and six rare CF variants (P5L, G85E, V232D, L1077P, W1098R, N1303K). A) The average Rosetta energy scores (± SEM) for the 100 lowest scoring models of the VX-445-bound state are plotted against those of the apo CFTR variant models. A reference line corresponding to no stabilization is shown for reference. All variants except for the non-responsive G85E fall below the line, which confirms VX-445 enhances stability. B) The total ΔRMSD of the active conformation of NBD2 is shown for variants bound to VX-445. Red bars show increasing deviations from the native NBD2 conformation in the mutant models and blue bars how much VX-445 suppresses these conformational defects in NBD2. C) Maps of the change in RMSD between G85E modeled with and without VX-445 shows which structural regions are stabilized by VX-445. Structurally variable regions in the ensemble shown in red, while areas adopting a more ordered conformation are shown in blue. VX-445 appears to primarily suppress conformational defects within the NBD2 region of this variant. D) Maps of the change in RMSD between L1077P modeled with and without VX-445 shows which structural regions are stabilized by VX-445. Structurally variable regions in the ensemble shown in red, while areas adopting a more ordered conformation are shown in blue. VX-445 appears to primarily suppress conformational defects within the NBD2 region of this variant. E) Maps of the change in RMSD between N1303K modeled with and without VX-445 shows that few structural regions are stabilized by VX-445 for N1303K, which responds poorly to VX-445 *in vitro*. Statistical significances were calculated using a non-parametric Wilcoxon signed-rank test compared to zero to determine if distributions changes were significantly different from zero, and p values were depicted by *< 0.05, **< 0.01, ***< 0.001, and ****< 0.0001. Error bars indicate the standard error of the mean.

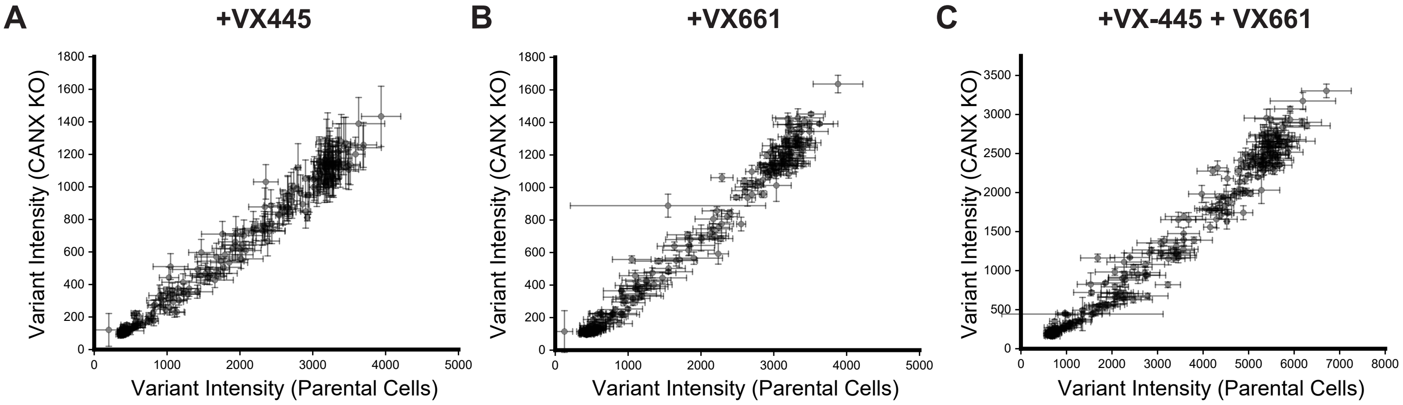

Figure S6. Influence of Correctors on the Plasma Membrane Expression in Parental and CANX Knock-out Cells. Deep mutational scanning measurements of the plasma membrane expression of CF variants in CANX knockout cells in the presence of A) 3 μM VX-445, B) 3 μM VX-661, or C) 3 μM VX-445 + 3 μM VX-661 are plotted against the corresponding variant measurements under identical conditions in the parental HEK-293T cell line. Values represent the average of three biological replicates and error bars represent the standard deviation. Trends are generally linear and there are few variants that stray from the tend, which suggests CF variants that respond in one cell line have a similar response in the other.

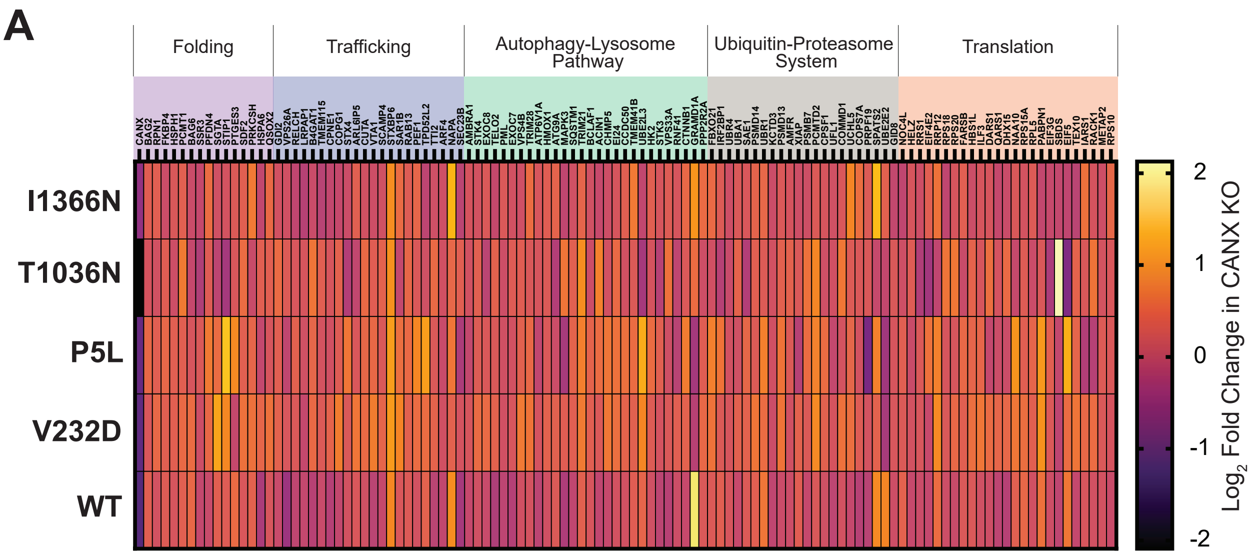

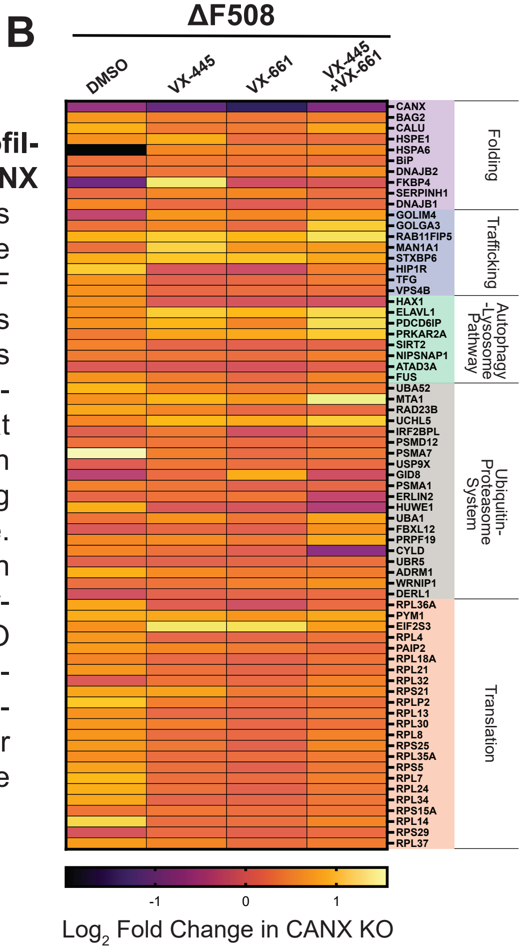

Figure S7. Comparative Interactome Profiling of CF variants in Parental and CANX Knockout HEK293T Cells. Proteomic mass spectrometry was used to compare the protein-protein interactions formed by CF variants in the context of CANX knockout cells relative to the parental cell line. A) Heat maps depict the $\log_2$ fold change in protein abundances of various classes of interactors that associate with each indicated CF variant in CANX KO cells relative to their corresponding abundance in the parental HEK293T cell line. B) Heat maps depict the $\log_2$ fold change in protein abundances of various classes of interactors that associate with ΔF508 in CANX KO cells treated with 3 μM of the indicated corrector(s) relative to their corresponding abundance in the parental HEK293T cell line under identical conditions. Protein interactors that are grouped according to their pathways.

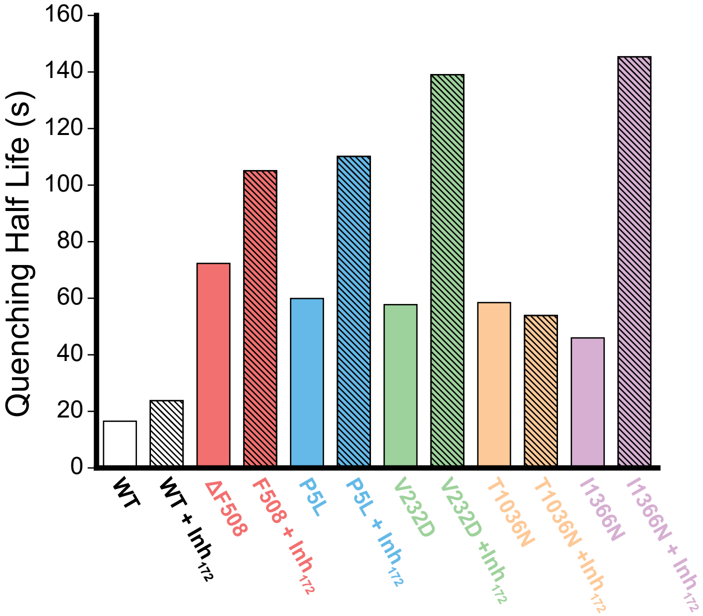

Figure S8. Impact of a CFTR-Specific Inhibitor on Observed hYFP Quenching Kinetics in Recombinant Cells Expressing CF Variants. The functional properties of CF variants are compared in various cells that feature endogenous CANX expression or deficient CANX expression under various experimental conditions. Bar graphs depict the fitted half-lives of the hYFP quenching reactions among cells expressing each indicated CF variant in parental HEK-293T cells treated with either vehicle (open bars) or with 10 μM of the CFTR-specific inhibitor-172 (Inh_{172}). A slowing of quenching was observed in the context of recombinant cell lines expressing each variant except T1036N. Though it is unclear why inhibition was not observed in this cell line, it is possible that this variant may have limited affinity for the inhibitor. Nevertheless, this cell line exhibits an intermediate quenching rate that lies between wild-type and ΔF508, which suggests the observed quencing is unlikely to be non-specific.

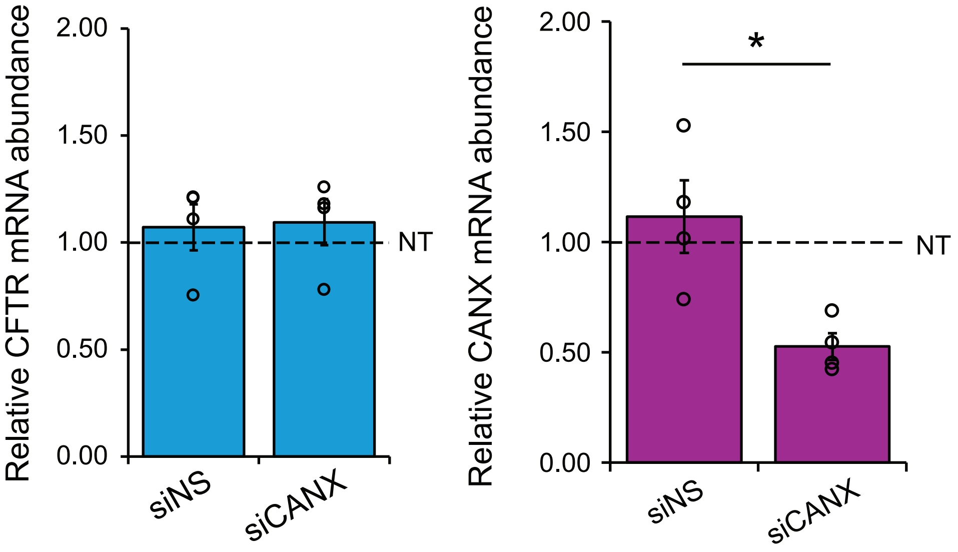

Figure S9. Validation of siRNA-Mediated CANX Knockdowns in FRT Cells . Quantitative reverse-transcriptase PCR was used to measure the relative abundance of the CFTR and CANX transcripts in FRT cells following transfection with either a non-specific or CANX-specific siRNA.ΔCT values for the target transcripts were normalized relative to the corresponding values of actin B transcript.

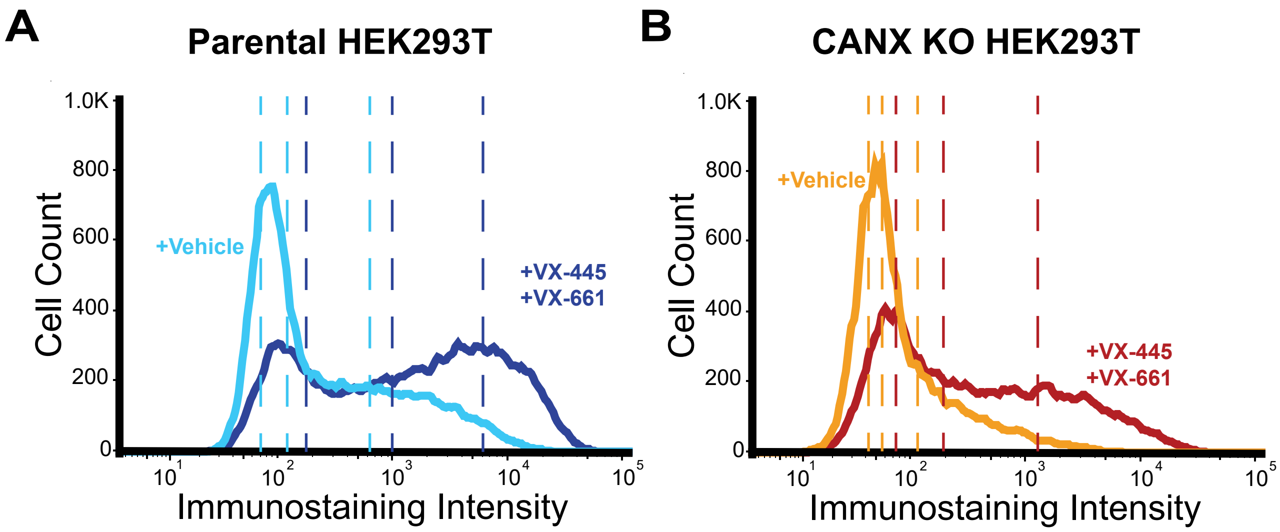

Figure S10. Comparison of Sorting Gates for Deep Mutational Scanning. Histograms depict the distribution of surface immunostaining intensities of recombinant libraries expressing CF variants in the A) parental and B) CANX knockout HEK293T cell lines. The approximate positions of the sorting gates used to fractionate each cell line into four equal quartiles based on immunostaining intensity are shown. Raw data and exact gating information for each experiment can be found in the Mendeley data directory.

Table S1. Deep Mutational Scanning Measurements for CF Variants in Treated Parental and CANX KO Cells

Variant	Amino Acid Substitution	CFTR sub-domain	Parental Surface Immuno-staining Intensity (Apo)	Parental Surface Immuno-staining Intensity (VX-661)	Parental Surface Immuno-staining Intensity (VX-445)	Parental Surface Immuno-staining Intensity (VX-445 + VX-661)	CANX KO Surface Immuno-staining Intensity (Apo)	CANX KO Surface Immuno-staining Intensity (VX-661)	CANX KO Surface Immuno-staining Intensity (VX-445)	CANX KO Surface Immuno-staining Intensity (VX-445 + VX-661)
**R3W**	7A>T	Lasso	913 ± 51	2602 ± 179	2934 ± 24	5281 ± 254	247 ± 9	980 ± 25	949 ± 119	2336 ± 48
**P5L**	14C>T	Lasso	260 ± 37	921 ± 235	2438 ± 152	4780 ± 95	78 ± 15	219 ± 19	774 ± 123	2015 ± 95
**S13F**	38C>T	Lasso	202 ± 43	498 ± 103	616 ± 51	2179 ± 291	72 ± 17	126 ± 13	169 ± 18	722 ± 73
**L15P**	44T>C	Lasso	212 ± 49	566 ± 126	535 ± 12	1845 ± 336	72 ± 19	126 ± 9	134 ± 11	538 ± 54
**G27R**	79G>A	Lasso	205 ± 43	518 ± 112	644 ± 52	2107 ± 395	70 ± 18	114 ± 21	154 ± 18	663 ± 24
**R31C**	91C>T	Lasso	532 ± 35	1853 ± 338	2224 ± 259	4364 ± 369	155 ± 6	680 ± 43	737 ± 66	1934 ± 50
**R31L**	92G>T	Lasso	352 ± 18	1219 ± 289	2343 ± 279	4274 ± 352	108 ± 11	378 ± 26	731 ± 86	1784 ± 11
**A46D**	137C>A	Lasso	218 ± 44	564 ± 128	1461 ± 213	3573 ± 348	70 ± 15	133 ± 11	455 ± 50	1474 ± 73
**E56K**	166G>A	Lasso	208 ± 48	825 ± 188	446 ± 42	1723 ± 325	72 ± 16	249 ± 16	115 ± 17	557 ± 23
**W57G**	169T>G	Lasso	200 ± 48	450 ± 85	392 ± 39	637 ± 109	67 ± 17	103 ± 15	102 ± 22	180 ± 40
**E60K**	178G>A	Lasso	198 ± 45	513 ± 117	388 ± 37	903 ± 161	66 ± 14	130 ± 18	100 ± 20	263 ± 16
**P67L**	200C>T	Lasso	212 ± 50	786 ± 146	447 ± 48	1782 ± 293	70 ± 15	225 ± 19	110 ± 16	557 ± 57
**R74W**	220C>T	Lasso	518 ± 20	1703 ± 233	1562 ± 220	3568 ± 322	129 ± 5	556 ± 49	443 ± 13	1274 ± 36
**R74Q**	221G>A	Lasso	784 ± 73	2975 ± 174	2175 ± 172	5170 ± 152	211 ± 8	1142 ± 35	722 ± 65	2387 ± 40
**R75Q**	224G>A	Lasso	759 ± 89	2886 ± 76	1938 ± 232	5045 ± 53	212 ± 6	1137 ± 9	662 ± 81	2330 ± 105
**G85E**	254G>A	TMD1	200 ± 43	454 ± 103	384 ± 42	676 ± 97	67 ± 15	109 ± 16	102 ± 17	184 ± 25
**G91R**	271G>A	TMD1	195 ± 47	528 ± 131	409 ± 38	1639 ± 312	67 ± 17	121 ± 13	105 ± 16	456 ± 35
**E92K**	274G>A	TMD1	204 ± 57	488 ± 89	405 ± 49	720 ± 93	70 ± 14	112 ± 15	105 ± 15	190 ± 27
**Q98R**	293A>G	TMD1	210 ± 47	951 ± 173	490 ± 21	2170 ± 408	69 ± 15	296 ± 23	124 ± 13	708 ± 94
**P99L**	296C>T	TMD1	428 ± 20	906 ± 212	1415 ± 211	1921 ± 357	118 ± 10	226 ± 8	355 ± 40	562 ± 30
**L102R**	305T>G	TMD1	199 ± 42	441 ± 96	381 ± 43	697 ± 161	64 ± 15	96 ± 15	90 ± 14	159 ± 28
**Y109N**	325T>A	TMD1	743 ± 93	2484 ± 77	1939 ± 188	4444 ± 160	211 ± 2	939 ± 13	641 ± 91	1941 ± 15
**D110Y**	328G>T	TMD1	495 ± 20	2140 ± 204	1594 ± 190	4397 ± 231	134 ± 5	721 ± 25	464 ± 44	1715 ± 55
**D110H**	328G>C	TMD1	1065 ± 79	3097 ± 102	2606 ± 163	5337 ± 143	274 ± 12	1111 ± 36	845 ± 75	2285 ± 59
**D110E**	330C>A	TMD1	1337 ± 46	3511 ± 195	3237 ± 85	5912 ± 341	393 ± 32	1452 ± 10	1197 ± 146	3071 ± 34
**E116K**	346G>A	TMD1	982 ± 92	2990 ± 37	2343 ± 223	4936 ± 68	284 ± 18	1195 ± 49	877 ± 116	2491 ± 79
**R117C**	349C>T	TMD1	833 ± 50	2405 ± 122	2310 ± 148	4593 ± 102	220 ± 2	841 ± 23	731 ± 90	1887 ± 45
**R117H**	350G>A	TMD1	1060 ± 65	2853 ± 51	2668 ± 56	4953 ± 141	272 ± 10	959 ± 21	839 ± 67	2010 ± 46
**R117P**	350G>C	TMD1	979 ± 109	2679 ± 132	2656 ± 93	4776 ± 74	299 ± 25	1023 ± 21	952 ± 118	2280 ± 51
**R117L**	350G>T	TMD1	762 ± 45	2191 ± 188	2080 ± 210	4156 ± 144	192 ± 1	701 ± 25	609 ± 57	1557 ± 63
**A120T**	358G>A	TMD1	1302 ± 43	3206 ± 142	3217 ± 57	5466 ± 178	346 ± 15	1212 ± 46	1046 ± 108	2453 ± 74
**G126D**	377G>A	TMD1	350 ± 26	1472 ± 330	1303 ± 157	3413 ± 340	99 ± 11	444 ± 33	374 ± 47	1313 ± 42
**I148T**	443T>C	TMD1	1049 ± 49	3134 ± 194	2588 ± 30	5261 ± 210	289 ± 20	1165 ± 29	874 ± 109	2401 ± 36
**Y161D**	481T>G	TMD1	199 ± 51	474 ± 108	427 ± 50	1665 ± 232	69 ± 16	120 ± 9	110 ± 18	535 ± 34
**Y161C**	482A>C	TMD1	206 ± 43	582 ± 121	749 ± 35	3240 ± 362	72 ± 17	142 ± 10	204 ± 23	1152 ± 78
**L165S**	494T>C	TMD1	199 ± 58	495 ± 104	806 ± 17	2573 ± 266	73 ± 15	126 ± 19	224 ± 34	955 ± 120
**R170H**	509G>A	TMD1	1216 ± 33	3030 ± 104	2953 ± 56	5204 ± 129	313 ± 12	1096 ± 10	952 ± 107	2198 ± 21
**L172I**	514C>A	TMD1	1116 ± 50	2953 ± 114	3109 ± 130	5344 ± 384	337 ± 9	1219 ± 79	1155 ± 138	2651 ± 129
**G178R**	532G>A	TMD1	1009 ± 92	2784 ± 73	2655 ± 101	4991 ± 172	267 ± 13	1025 ± 26	875 ± 85	2224 ± 21
**G178E**	533G>A	TMD1	1369 ± 32	3348 ± 165	3392 ± 128	5817 ± 362	391 ± 20	1369 ± 23	1277 ± 167	2892 ± 95
**F191V**	571T>G	TMD1	233 ± 42	787 ± 168	1526 ± 206	3575 ± 369	77 ± 16	220 ± 9	466 ± 65	1397 ± 73
**D192G**	575A>G	TMD1	251 ± 39	753 ± 194	1774 ± 241	3356 ± 364	78 ± 15	172 ± 12	506 ± 53	1160 ± 51
**E193K**	577G>A	TMD1	838 ± 57	2607 ± 136	2426 ± 94	4887 ± 101	243 ± 14	1003 ± 18	885 ± 97	2346 ± 50

Variant	Amino Acid Substitution	CFTR sub-domain	Parental Surface Immuno-staining Intensity (Apo)	Parental Surface Immuno-staining Intensity (VX-661)	Parental Surface Immuno-staining Intensity (VX-445)	Parental Surface Immuno-staining Intensity (VX-445 + VX-661)	CANX KO Surface Immuno-staining Intensity (Apo)	CANX KO Surface Immuno-staining Intensity (VX-661)	CANX KO Surface Immuno-staining Intensity (VX-445)	CANX KO Surface Immuno-staining Intensity (VX-445 + VX-661)
**G194R**	580G>A	TMD1	765 ± 93	2159 ± 269	2537 ± 190	4477 ± 196	201 ± 7	746 ± 33	832 ± 99	1832 ± 100
**G194V**	581G>T	TMD1	303 ± 36	1255 ± 286	1233 ± 194	3342 ± 464	89 ± 12	422 ± 54	352 ± 52	1257 ± 36
**H199Y**	595C>T	TMD1	201 ± 45	454 ± 109	391 ± 47	689 ± 122	70 ± 16	111 ± 12	104 ± 20	188 ± 27
**V201M**	601G>A	TMD1	496 ± 15	2282 ± 166	1701 ± 262	4523 ± 37	123 ± 11	776 ± 88	468 ± 34	1631 ± 99
**P205S**	613C>T	TMD1	205 ± 41	478 ± 97	437 ± 22	841 ± 154	71 ± 14	123 ± 15	112 ± 23	245 ± 16
**L206W**	617T>G	TMD1	203 ± 46	618 ± 81	556 ± 19	1586 ± 203	67 ± 14	151 ± 19	138 ± 17	454 ± 18
**L227R**	680T>G	TMD1	200 ± 48	476 ± 121	401 ± 72	657 ± 157	70 ± 14	118 ± 11	105 ± 18	197 ± 32
**V232D**	695T>A	TMD1	202 ± 47	513 ± 113	446 ± 35	1117 ± 209	69 ± 13	120 ± 10	108 ± 21	272 ± 11
**A234D**	701C>A	TMD1	423 ± 24	1266 ± 281	1549 ± 310	3080 ± 417	124 ± 7	390 ± 21	495 ± 92	1219 ± 31
**Q237E**	709C>G	TMD1	1299 ± 68	3076 ± 72	3282 ± 109	5328 ± 189	347 ± 17	1129 ± 22	1127 ± 119	2377 ± 47
**Q237H**	711G>C	TMD1	1339 ± 34	3401 ± 162	3487 ± 166	5954 ± 306	343 ± 19	1229 ± 36	1131 ± 112	2678 ± 90
**R258G**	772A>G	TMD1	239 ± 56	663 ± 138	749 ± 41	1551 ± 227	77 ± 15	148 ± 22	173 ± 20	440 ± 33
**M265R**	794T>G	TMD1	223 ± 41	568 ± 185	571 ± 33	1069 ± 146	75 ± 15	130 ± 19	146 ± 17	325 ± 7
**F311L**	933C>G	TMD1	1199 ± 42	2912 ± 94	3141 ± 26	5260 ± 110	324 ± 12	1094 ± 13	1047 ± 93	2352 ± 21
	933C>A	TMD1	1194 ± 40	2901 ± 89	3169 ± 22	5428 ± 106	354 ± 19	1197 ± 20	1165 ± 140	2637 ± 116
**G314E**	941G>A	TMD1	894 ± 89	2598 ± 98	2703 ± 150	5091 ± 116	265 ± 10	1041 ± 17	975 ± 115	2489 ± 63
**L320V**	958T>G	TMD1	1401 ± 21	3491 ± 325	3425 ± 186	5637 ± 494	405 ± 24	1389 ± 26	1251 ± 176	2711 ± 80
**R334W**	1000C>T	TMD1	1429 ± 50	3421 ± 211	3339 ± 136	5746 ± 483	395 ± 25	1407 ± 28	1214 ± 148	2838 ± 65
**R334L**	1001G>T	TMD1	219 ± 51	555 ± 111	602 ± 25	1298 ± 236	76 ± 15	143 ± 13	148 ± 13	342 ± 13
**R334Q**	1001G>A	TMD1	1310 ± 76	2985 ± 91	3277 ± 87	5342 ± 243	363 ± 23	1127 ± 10	1153 ± 144	2454 ± 53
**I336K**	1007T>A	TMD1	203 ± 44	537 ± 109	469 ± 33	1194 ± 229	73 ± 18	130 ± 17	125 ± 14	351 ± 19
**T338I**	1013C>T	TMD1	1194 ± 45	3206 ± 193	3098 ± 100	5322 ± 316	327 ± 24	1177 ± 21	1080 ± 116	2466 ± 83
**S341P**	1021T>C	TMD1	363 ± 18	1354 ± 250	1290 ± 164	3454 ± 370	100 ± 13	402 ± 29	340 ± 29	1167 ± 66
**L346P**	1037T>C	TMD1	188 ± 52	463 ± 84	387 ± 44	1144 ± 184	67 ± 13	114 ± 11	99 ± 13	321 ± 9
**R347H**	1040G>A	TMD1	1401 ± 55	3350 ± 219	3697 ± 180	5851 ± 609	368 ± 20	1182 ± 19	1258 ± 138	2658 ± 17
**R347P**	1040G>C	TMD1	239 ± 39	877 ± 182	1104 ± 141	2784 ± 445	72 ± 15	163 ± 20	228 ± 29	676 ± 34
	1040G>T	TMD1	858 ± 59	2237 ± 140	2925 ± 28	4885 ± 206	193 ± 10	592 ± 63	811 ± 20	1742 ± 89
**A349V**	1046C>T	TMD1	1036 ± 72	3029 ± 63	2664 ± 100	5321 ± 122	261 ± 10	1082 ± 28	866 ± 60	2274 ± 55
**R352W**	1054C>T	TMD1	511 ± 23	1636 ± 235	1470 ± 174	3457 ± 385	168 ± 7	639 ± 26	597 ± 80	1653 ± 95
**R352Q**	1055G>A	TMD1	1424 ± 63	3292 ± 82	3515 ± 134	5674 ± 277	367 ± 16	1132 ± 37	1138 ± 125	2350 ± 15
**Q359K/ T360K**	1075-1079C>A	TMD1	327 ± 23	1184 ± 267	1583 ± 235	3463 ± 393	98 ± 13	337 ± 25	450 ± 51	1197 ± 44
**Q359R**	1076A>G	TMD1	1184 ± 66	2943 ± 121	3011 ± 58	5257 ± 169	316 ± 18	1102 ± 39	998 ± 93	2317 ± 40
**W361R**	1081T>C	TMD1	194 ± 49	435 ± 87	427 ± 64	738 ± 80	69 ± 15	106 ± 16	110 ± 13	203 ± 29
**S364P**	1090T>C	TMD1	209 ± 43	457 ± 103	423 ± 42	708 ± 113	67 ± 15	108 ± 13	101 ± 15	184 ± 25
**G404R**	1210G>C		1312 ± 42	3342 ± 232	3212 ± 84	5476 ± 342	372 ± 19	1268 ± 8	1131 ± 134	2576 ± 75
**D443Y**	1327G>T	NBD1	362 ± 15	1162 ± 276	1071 ± 154	2736 ± 419	121 ± 5	378 ± 14	342 ± 34	1079 ± 40
**L453S**	1358T>C	NBD1	244 ± 85	527 ± 43	499 ± 182	948 ± 153	66 ± 12	112 ± 12	115 ± 23	255 ± 25
**A455E**	1364C>A	NBD1	197 ± 50	482 ± 140	400 ± 63	726 ± 95	67 ± 17	101 ± 10	97 ± 15	185 ± 37
**V456F**	1366G>T	NBD1	203 ± 44	426 ± 95	408 ± 30	777 ± 116	67 ± 15	103 ± 12	101 ± 15	215 ± 24
**V456A**	1367T>C	NBD1	198 ± 51	460 ± 91	398 ± 46	756 ± 91	71 ± 19	120 ± 12	105 ± 16	210 ± 43
**G463D**	1388G>T	NBD1	200 ± 33	515 ± 119	731 ± 74	2288 ± 392	69 ± 14	119 ± 18	180 ± 22	705 ± 81
**L467P**	1400T>C	NBD1	189 ± 48	410 ± 95	367 ± 50	640 ± 87	67 ± 14	104 ± 10	95 ± 16	180 ± 34
**M470V**	1408A>G	NBD1	1315 ± 63	2987 ± 143	2798 ± 36	4316 ± 210	387 ± 29	1256 ± 35	1119 ± 147	2317 ± 88
**E474K**	1420G>A	NBD1	196 ± 44	434 ± 92	452 ± 21	1269 ± 283	67 ± 13	105 ± 14	109 ± 14	363 ± 6
**G480S**	1438G>T	NBD1	206 ± 41	450 ± 101	438 ± 21	1050 ± 168	71 ± 18	113 ± 14	118 ± 18	296 ± 29
**S492F**	1475C>T	NBD1	195 ± 50	428 ± 91	374 ± 35	734 ± 105	67 ± 16	107 ± 15	100 ± 14	196 ± 19
**I502T**	1505T>C	NBD1	199 ± 43	426 ± 81	375 ± 57	700 ± 156	68 ± 16	109 ± 15	105 ± 16	208 ± 31

Variant	Amino Acid Substitution	CFTR sub-domain	Parental Surface Immuno-staining Intensity (Apo)	Parental Surface Immuno-staining Intensity (VX-661)	Parental Surface Immuno-staining Intensity (VX-445)	Parental Surface Immuno-staining Intensity (VX-445 + VX-661)	CANX KO Surface Immuno-staining Intensity (Apo)	CANX KO Surface Immuno-staining Intensity (VX-661)	CANX KO Surface Immuno-staining Intensity (VX-445)	CANX KO Surface Immuno-staining Intensity (VX-445 + VX-661)
**F508C**	1523T>G	NBD1	1015 ± 78	2970 ± 108	3056 ± 46	5590 ± 283	263 ± 12	1090 ± 30	964 ± 85	2480 ± 53
**D513G**	1538A>G	NBD1	199 ± 46	449 ± 101	404 ± 39	736 ± 124	69 ± 15	108 ± 14	103 ± 20	213 ± 30
**V520F**	1558G>T	NBD1	201 ± 49	445 ± 98	380 ± 48	681 ± 131	67 ± 16	107 ± 13	97 ± 14	192 ± 30
**E528E**	1584G>A	NBD1	1349 ± 57	3303 ± 171	3281 ± 85	5641 ± 349	345 ± 18	1148 ± 32	1054 ± 106	2414 ± 33
**S549N**	1646G>A	NBD1	1340 ± 53	3197 ± 138	3227 ± 62	5604 ± 178	379 ± 14	1311 ± 40	1179 ± 134	2792 ± 86
**S549R**	1645A>C	NBD1	404 ± 21	1097 ± 215	1027 ± 133	2265 ± 376	147 ± 9	458 ± 33	442 ± 65	1110 ± 117
	1647T>A	NBD1	415 ± 9	1108 ± 204	1073 ± 120	2264 ± 339	131 ± 5	393 ± 20	366 ± 42	914 ± 55
	1647T>G	NBD1	385 ± 10	1068 ± 165	1036 ± 80	2251 ± 567	105 ± 15	291 ± 17	256 ± 44	661 ± 88
**G551S**	1651G>A	NBD1	1483 ± 66	3627 ± 252	3690 ± 250	6283 ± 509	398 ± 12	1391 ± 9	1241 ± 135	2855 ± 34
**G551D**	1652G>A	NBD1	1429 ± 52	3489 ± 257	3588 ± 129	6172 ± 428	384 ± 16	1344 ± 47	1201 ± 104	2900 ± 48
**R553N**	1658G>A	NBD1	1334 ± 31	3197 ± 156	3179 ± 148	5442 ± 153	371 ± 22	1251 ± 31	1112 ± 119	2594 ± 72
**A554E**	(1661C>A)	NBD1	329 ± 37	968 ± 175	918 ± 73	2063 ± 298	115 ± 8	313 ± 26	313 ± 35	792 ± 34
**L558S**	1673T>C	NBD1	193 ± 47	443 ± 72	365 ± 41	639 ± 114	67 ± 14	103 ± 14	99 ± 16	170 ± 29
**A559T**	1675G>A	NBD1	199 ± 51	440 ± 82	398 ± 46	659 ± 81	66 ± 17	102 ± 17	95 ± 20	160 ± 21
**R560K**	1679G>A	NBD1	196 ± 51	425 ± 96	368 ± 39	614 ± 109	66 ± 15	97 ± 13	91 ± 19	170 ± 25
**R560T**	1679G>C	NBD1	202 ± 49	468 ± 111	396 ± 27	669 ± 91	66 ± 14	104 ± 14	97 ± 18	187 ± 33
**R560S**	1680A>C	NBD1	192 ± 49	448 ± 119	382 ± 46	666 ± 94	70 ± 17	108 ± 10	103 ± 19	189 ± 32
**A561E**	1682C>A	NBD1	194 ± 51	410 ± 71	376 ± 45	659 ± 79	69 ± 17	117 ± 17	107 ± 13	193 ± 25
**V562I**	1684G>A	NBD1	1057 ± 67	2698 ± 128	2646 ± 52	4692 ± 233	286 ± 1	958 ± 48	877 ± 85	2016 ± 35
**Y563N**	1687T>A	NBD1	193 ± 50	423 ± 85	364 ± 44	656 ± 63	71 ± 23	107 ± 20	98 ± 29	196 ± 44
**Y563D**	1687T>G	NBD1	193 ± 50	452 ± 127	388 ± 40	654 ± 81	69 ± 13	107 ± 18	103 ± 12	192 ± 31
**Y569D**	1705T>G	NBD1	206 ± 51	457 ± 90	408 ± 42	682 ± 99	69 ± 17	114 ± 13	103 ± 19	197 ± 40
**P574H**	1721C>A	NBD1	203 ± 50	475 ± 109	440 ± 45	1047 ± 184	69 ± 17	110 ± 14	118 ± 19	303 ± 12
**F575Y**	1724T>A	NBD1	227 ± 49	571 ± 130	567 ± 23	1353 ± 213	81 ± 11	161 ± 13	171 ± 17	494 ± 20
**G576A**	1727G>C	NBD1	1400 ± 58	3318 ± 138	3352 ± 92	5659 ± 291	383 ± 15	1297 ± 55	1152 ± 103	2685 ± 30
**D579G**	1736A>G	NBD1	398 ± 18	1102 ± 213	1000 ± 98	2227 ± 304	123 ± 9	378 ± 6	351 ± 34	902 ± 84
**E588V**	1763A>T	NBD1	417 ± 8	1136 ± 253	1009 ± 123	2088 ± 333	124 ± 8	346 ± 39	316 ± 39	713 ± 44
**S589T**	1766G>C	NBD1	1170 ± 92	2920 ± 37	2832 ± 21	4928 ± 129	312 ± 7	1073 ± 23	963 ± 110	2163 ± 50
**I601F**	1801A>T	NBD1	236 ± 47	644 ± 156	550 ± 34	1089 ± 165	79 ± 17	142 ± 23	142 ± 16	330 ± 16
**H609R**	1826A>G	NBD1	201 ± 45	470 ± 93	421 ± 54	768 ± 123	72 ± 19	107 ± 19	100 ± 17	199 ± 24
**A613T**	1837G>A	NBD1	208 ± 46	476 ± 98	413 ± 43	773 ± 126	69 ± 13	110 ± 20	108 ± 14	209 ± 28
**D614G**	1841A>G	NBD1	211 ± 55	538 ± 123	448 ± 35	948 ± 139	74 ± 18	127 ± 12	126 ± 16	295 ± 9
**I618T**	1853T>C	NBD1	209 ± 46	488 ± 110	471 ± 52	994 ± 192	69 ± 14	110 ± 12	115 ± 14	270 ± 11
**G622D**	1865G>A	NBD1	232 ± 40	723 ± 187	906 ± 75	2722 ± 328	77 ± 15	185 ± 5	263 ± 44	974 ± 81
**G628R**	1882G>A	NBD1	203 ± 54	489 ± 143	419 ± 44	812 ± 145	69 ± 16	113 ± 13	110 ± 16	234 ± 30
	1882G>C	NBD1	196 ± 50	443 ± 79	398 ± 47	760 ± 138	73 ± 16	119 ± 18	122 ± 21	252 ± 32
**R668C**	2002C>T	R Domain	1210 ± 61	2968 ± 149	2900 ± 39	5071 ± 152	339 ± 11	1144 ± 10	1051 ± 114	2482 ± 64
**S737F**	2210C>T	R Domain	1365 ± 46	3295 ± 83	3273 ± 39	5655 ± 202	373 ± 7	1287 ± 33	1141 ± 108	2608 ± 18
**P750L**	2249C>T	R Domain	363 ± 13	1192 ± 292	1228 ± 219	2757 ± 423	104 ± 11	332 ± 17	341 ± 36	940 ± 19
**R751L**	2252G>T	R Domain	1199 ± 34	3102 ± 89	3010 ± 57	5095 ± 211	334 ± 21	1147 ± 35	1045 ± 110	2386 ± 59
**V754M**	2260G>A	R Domain	1316 ± 53	3192 ± 165	3189 ± 147	5394 ± 490	403 ± 30	1421 ± 37	1260 ± 156	2954 ± 114
**I807M**	2374C>G	R Domain	1329 ± 63	3229 ± 153	3251 ± 150	5529 ± 300	370 ± 21	1249 ± 26	1136 ± 115	2625 ± 55
**E822K**	2464G>A	R Domain	636 ± 56	1830 ± 354	2184 ± 142	4064 ± 249	179 ± 2	614 ± 34	745 ± 104	1796 ± 79
**D836Y**	2506G>T		1050 ± 50	2749 ± 112	2715 ± 111	4808 ± 100	295 ± 14	1017 ± 22	915 ± 107	2139 ± 71
**S912L**	2735C>T	TMD2	939 ± 34	2289 ± 152	2356 ± 168	4212 ± 345	313 ± 13	1060 ± 24	1031 ± 106	2275 ± 54
**D924N**	2770G>A	TMD2	1312 ± 37	3331 ± 349	3630 ± 357	6192 ± 717	394 ± 8	1428 ± 55	1388 ± 161	3173 ± 109
**L927P**	2780T>C	TMD2	675 ± 66	1813 ± 277	1760 ± 287	3579 ± 408	218 ± 6	709 ± 19	710 ± 78	1694 ± 44
**R933G**	2797A>G	TMD2	1617 ± 61	3882 ± 341	3938 ± 269	6709 ± 547	471 ± 14	1636 ± 54	1433 ± 186	3302 ± 87

Variant	Amino Acid Substitution	CFTR sub-domain	Parental Surface Immuno-staining Intensity (Apo)	Parental Surface Immuno-staining Intensity (VX-661)	Parental Surface Immuno-staining Intensity (VX-445)	Parental Surface Immuno-staining Intensity (VX-445 + VX-661)	CANX KO Surface Immuno-staining Intensity (Apo)	CANX KO Surface Immuno-staining Intensity (VX-661)	CANX KO Surface Immuno-staining Intensity (VX-445)	CANX KO Surface Immuno-staining Intensity (VX-445 + VX-661)
**H939R**	2816A>G	TMD2	1285 ± 63	3413 ± 85	3494 ± 135	5857 ± 264	337 ± 18	1145 ± 18	1106 ± 123	2573 ± 39
**S945L**	2834C>T	TMD2	284 ± 37	997 ± 201	1163 ± 112	2738 ± 306	86 ± 12	253 ± 10	303 ± 29	941 ± 68
**M952T**	2855T>C	TMD2	1241 ± 51	3020 ± 48	3115 ± 39	5421 ± 252	352 ± 20	1229 ± 10	1126 ± 142	2580 ± 72
**M952I**	2856G>A	TMD2	916 ± 99	2552 ± 61	2903 ± 68	5042 ± 182	221 ± 6	775 ± 42	849 ± 102	1989 ± 37
**L967S**	2900T>C	TMD2	728 ± 71	1902 ± 393	1852 ± 255	3415 ± 399	177 ± 3	566 ± 38	534 ± 67	1230 ± 46
**G970R**	2908G>C	TMD2	1358 ± 51	3228 ± 234	3225 ± 151	5314 ± 278	408 ± 22	1387 ± 25	1221 ± 141	2743 ± 126
**G970D**	2909G>A	TMD2	1160 ± 74	2821 ± 45	2738 ± 66	4828 ± 147	329 ± 24	1136 ± 16	1000 ± 110	2413 ± 105
**S977F**	2930C>T	TMD2	1218 ± 96	3010 ± 95	3276 ± 82	5622 ± 261	328 ± 18	1119 ± 47	1128 ± 124	2742 ± 130
**I980K**	2939T>A	TMD2	224 ± 39	605 ± 135	667 ± 34	1348 ± 216	73 ± 15	134 ± 12	150 ± 15	378 ± 3
**L997F**	2991G>C	TMD2	1297 ± 64	3275 ± 112	3451 ± 125	5910 ± 374	370 ± 16	1342 ± 28	1270 ± 158	2934 ± 83
**Y1014C**	3041A>G	TMD2	1139 ± 92	3111 ± 41	3083 ± 19	5309 ± 97	341 ± 22	1198 ± 37	1097 ± 124	2535 ± 93
**F1016S**	3047T>C	TMD2	361 ± 8	1334 ± 322	1677 ± 295	3682 ± 376	118 ± 9	475 ± 31	569 ± 85	1650 ± 93
**I1027T**	3080T>C	TMD2	1030 ± 80	2640 ± 108	2540 ± 50	4681 ± 132	270 ± 8	936 ± 51	824 ± 79	1970 ± 12
**Y1032C**	3095A>G	TMD2	234 ± 51	758 ± 242	1944 ± 177	3971 ± 288	82 ± 15	209 ± 10	712 ± 106	1982 ± 110
**T1036N**	3107C>A	TMD2	214 ± 66	587 ± 71	1135 ± 98	3228 ± 275	65 ± 15	124 ± 15	231 ± 29	820 ± 36
**F1052V**	3154T>G	TMD2	1248 ± 48	3204 ± 151	3205 ± 152	5471 ± 335	391 ± 33	1387 ± 51	1282 ± 173	2945 ± 125
**T1053I**	3158C>T	TMD2	1302 ± 56	3122 ± 105	3074 ± 72	5343 ± 109	333 ± 15	1163 ± 10	999 ± 100	2378 ± 50
**H1054D**	3160C>G	TMD2	207 ± 52	557 ± 107	1086 ± 139	3412 ± 304	69 ± 16	124 ± 16	293 ± 38	1215 ± 82
**K1060T**	3179A>C	TMD2	1129 ± 59	2870 ± 115	2783 ± 83	4958 ± 59	323 ± 18	1111 ± 10	992 ± 127	2331 ± 42
**G1061R**	3181G>C	TMD2	202 ± 42	469 ± 76	546 ± 57	1709 ± 321	69 ± 15	106 ± 16	139 ± 14	511 ± 28
**R1066C**	3196C>T	TMD2	194 ± 50	426 ± 68	363 ± 66	681 ± 106	67 ± 14	96 ± 12	94 ± 17	169 ± 30
**R1066H**	3197G>A	TMD2	207 ± 43	508 ± 112	834 ± 88	2822 ± 553	80 ± 18	152 ± 14	276 ± 29	1225 ± 126
**A1067T**	3199G>A	TMD2	747 ± 45	2361 ± 137	2405 ± 212	4870 ± 92	191 ± 6	820 ± 35	727 ± 75	2007 ± 50
**G1069R**	3205G>A	TMD2	1330 ± 73	3170 ± 138	3182 ± 19	5586 ± 205	335 ± 18	1135 ± 25	1013 ± 97	2344 ± 14
**R1070W**	3208C>T	TMD2	431 ± 25	1553 ± 331	1656 ± 253	3800 ± 405	120 ± 11	482 ± 11	427 ± 46	1390 ± 41
**R1070Q**	3209G>A	TMD2	1402 ± 54	3379 ± 169	3362 ± 133	5549 ± 317	395 ± 20	1344 ± 34	1221 ± 167	2734 ± 85
**F1074L**	3222T>G	TMD2	371 ± 11	1557 ± 229	1998 ± 205	4373 ± 180	107 ± 8	502 ± 32	618 ± 79	1860 ± 56
**L1077P**	3230T>C	TMD2	205 ± 46	458 ± 90	557 ± 17	2067 ± 314	68 ± 17	102 ± 16	144 ± 14	662 ± 59
**H1085P**	3254A>C	TMD2	202 ± 45	449 ± 84	423 ± 17	941 ± 123	69 ± 16	111 ± 18	110 ± 17	252 ± 20
**H1085R**	3254A>G	TMD2	214 ± 42	557 ± 139	803 ± 66	2258 ± 382	69 ± 17	123 ± 15	190 ± 22	639 ± 10
**W1098R**	3292T>C	TMD2	210 ± 47	481 ± 102	404 ± 46	676 ± 147	72 ± 17	129 ± 12	111 ± 18	207 ± 41
**W1098C**	3294G>C	TMD2	209 ± 45	550 ± 125	818 ± 64	2465 ± 422	68 ± 15	122 ± 12	175 ± 29	626 ± 39
	3294G>T	TMD2	221 ± 38	650 ± 204	818 ± 87	2293 ± 377	88 ± 23	189 ± 20	271 ± 34	880 ± 61
**F1099L**	3297C>A	TMD2	288 ± 31	1067 ± 234	2032 ± 263	4356 ± 177	90 ± 12	318 ± 4	643 ± 99	1771 ± 100
**M1101K**	3302T>A	TMD2	202 ± 47	463 ± 126	406 ± 45	740 ± 158	69 ± 15	122 ± 9	109 ± 20	211 ± 36
**M1101R**	3302T>G	TMD2	223 ± 30	509 ± 174	412 ± 51	745 ± 234	80 ± 14	152 ± 7	132 ± 21	271 ± 25
**S1118F**	3353C>T	TMD2	499 ± 46	1660 ± 291	2015 ± 303	4293 ± 457	133 ± 5	551 ± 34	557 ± 59	1658 ± 112
**I1139V**	3415A>G	TMD2	1222 ± 60	3124 ± 128	3023 ± 65	5157 ± 227	342 ± 22	1181 ± 39	1052 ± 134	2434 ± 175
**D1152H**	3454G>C	TMD2	1177 ± 59	3034 ± 205	2861 ± 77	5283 ± 400	293 ± 6	1012 ± 98	893 ± 50	2031 ± 171
**V1153E**	3458T>A	TMD2	302 ± 34	804 ± 170	980 ± 77	1858 ± 269	93 ± 20	203 ± 32	246 ± 26	566 ± 33
**L1156F**	3600G>T-3468G>T	TMD2	1248 ± 53	3224 ± 138	3263 ± 95	5550 ± 398	339 ± 16	1203 ± 2	1139 ± 142	2585 ± 71
**S1159P**	3475T>C	TMD2	1092 ± 39	2850 ± 96	3265 ± 131	5485 ± 328	286 ± 4	1046 ± 42	1076 ± 138	2493 ± 81
**S1159F**	3476C>T	TMD2	790 ± 71	2275 ± 162	2381 ± 161	15 ± 126	187 ± 3	686 ± 17	667 ± 91	1736 ± 13
**R1162L**	3485G>T	NBD2	828 ± 88	2243 ± 150	2338 ± 176	4138 ± 273	217 ± 7	754 ± 21	752 ± 82	1772 ± 4
**I1234V**	3700A>G	NBD2	1331 ± 52	3332 ± 189	3353 ± 128	5680 ± 377	362 ± 19	1303 ± 21	1157 ± 140	2659 ± 26
**S1235R**	3705T>G	NBD2	1345 ± 59	3405 ± 192	3378 ± 119	5751 ± 320	361 ± 14	1246 ± 33	1106 ± 108	2593 ± 91
**V1240G**	3719T>G	NBD2	671 ± 157	1840 ± 350	1793 ± 387	3072 ± 524	164 ± 14	644 ± 59	583 ± 95	1356 ± 49

Variant	Amino Acid Substitution	CFTR sub-domain	Parental Surface Immuno-staining Intensity (Apo)	Parental Surface Immuno-staining Intensity (VX-661)	Parental Surface Immuno-staining Intensity (VX-445)	Parental Surface Immuno-staining Intensity (VX-445 + VX-661)	CANX KO Surface Immuno-staining Intensity (Apo)	CANX KO Surface Immuno-staining Intensity (VX-661)	CANX KO Surface Immuno-staining Intensity (VX-445)	CANX KO Surface Immuno-staining Intensity (VX-445 + VX-661)
**G1244E**	3731G>A	NBD2	1352 ± 23	3364 ± 206	3353 ± 184	5631 ± 384	375 ± 21	1290 ± 11	1176 ± 130	2727 ± 97
**T1246I**	3737C>T	NBD2	1293 ± 59	3253 ± 144	3286 ± 87	5633 ± 307	335 ± 11	1133 ± 45	1044 ± 109	2439 ± 72
**G1249R**	3745G>A	NBD2	1220 ± 51	3155 ± 176	3087 ± 75	5352 ± 348	357 ± 20	1248 ± 38	1153 ± 154	2727 ± 141
**S1251N**	3752G>A	NBD2	1353 ± 41	3290 ± 176	3313 ± 165	5769 ± 330	371 ± 17	1276 ± 8	1146 ± 129	2654 ± 26
**S1255P**	3763T>C	NBD2	1258 ± 54	3153 ± 173	3161 ± 89	5435 ± 299	318 ± 15	1136 ± 36	1025 ± 121	2374 ± 16
**I1269N**	3806T>A	NBD2	554 ± 106	1420 ± 324	1419 ± 344	2391 ± 432	149 ± 4	547 ± 16	492 ± 47	1169 ± 7
**D1270N**	3808G>A	NBD2	1242 ± 88	3122 ± 51	3151 ± 42	5479 ± 180	342 ± 12	1199 ± 29	1093 ± 122	2573 ± 44
**W1282R**	3844T>C	NBD2	363 ± 35	925 ± 264	949 ± 230	1551 ± 393	110 ± 16	326 ± 32	302 ± 43	720 ± 22
**R1283M**	3848G>T	NBD2	422 ± 59	1132 ± 308	1151 ± 270	1855 ± 418	122 ± 16	398 ± 38	375 ± 42	843 ± 15
**R1283S**	3849G>C	NBD2	465 ± 82	1252 ± 336	1220 ± 290	2064 ± 431	130 ± 15	430 ± 15	414 ± 52	982 ± 43
**Q1291R**	3872A>G	NBD2	1389 ± 70	3474 ± 173	3453 ± 110	5869 ± 326	361 ± 16	1275 ± 15	1120 ± 131	2516 ± 78
**Q1291H**	3873G>C	NBD2	1280 ± 62	3203 ± 104	3235 ± 86	5568 ± 203	327 ± 14	1083 ± 25	1018 ± 111	2285 ± 55
**V1293G**	3878T>G	NBD2	1331 ± 61	3329 ± 164	3314 ± 47	5581 ± 117	363 ± 14	1250 ± 23	1119 ± 128	2487 ± 47
**N1303K**	3909C>G	NBD2	262 ± 34	630 ± 154	609 ± 58	1034 ± 219	88 ± 15	226 ± 6	206 ± 30	432 ± 5
**L1324P**	3971T>C	NBD2	249 ± 44	579 ± 124	548 ± 31	975 ± 211	91 ± 14	223 ± 7	210 ± 16	441 ± 18
**L1335P**	4004T>C	NBD2	243 ± 30	623 ± 151	564 ± 57	967 ± 168	97 ± 15	224 ± 25	224 ± 13	458 ± 5
**G1349D**	4046G>A	NBD2	1225 ± 38	3029 ± 77	3062 ± 59	5304 ± 214	338 ± 20	1152 ± 46	1060 ± 115	2437 ± 57
**I1366N**	4097T>A	NBD2	408 ± 59	1050 ± 265	1048 ± 239	1685 ± 374	148 ± 11	558 ± 22	510 ± 79	1163 ± 50
**H1375P**	4124A>C		1253 ± 69	3167 ± 42	3171 ± 66	5497 ± 218	353 ± 31	1287 ± 10	1171 ± 126	2767 ± 134
**L1480P**	4439T>C		1080 ± 93	2698 ± 85	2658 ± 60	4535 ± 131	295 ± 12	1098 ± 38	941 ± 123	2181 ± 84
**L138ins**	413_415dupTAC	TMD1	201 ± 49	572 ± 115	486 ± 40	2039 ± 293	75 ± 14	171 ± 20	142 ± 18	579 ± 25
**F312DEL**	935_937del	NBD1	647 ± 77	2008 ± 224	2329 ± 124	4508 ± 98	164 ± 2	683 ± 24	762 ± 87	1940 ± 156
**I507del**	1519_1521delATC	NBD1	199 ± 51	425 ± 87	365 ± 50	626 ± 89	75 ± 18	123 ± 14	112 ± 21	211 ± 29
**F508del**	1521_1523delCTT	NBD1	195 ± 47	453 ± 108	410 ± 47	891 ± 150	68 ± 15	110 ± 12	110 ± 16	256 ± 26
**T854T**	2562T>A		1294 ± 51	3240 ± 145	3213 ± 88	5519 ± 358	357 ± 10	1216 ± 17	1072 ± 125	2486 ± 54
	2562T>C		1329 ± 72	3214 ± 194	3270 ± 21	5657 ± 238	350 ± 18	1193 ± 54	1063 ± 117	2478 ± 68
	2562T>G		1287 ± 53	3169 ± 189	3164 ± 147	5401 ± 312	364 ± 14	1251 ± 44	1134 ± 116	2588 ± 72
**Q966=**	2988G>A	TMD2	1324 ± 43	3171 ± 147	3196 ± 88	5527 ± 156	332 ± 19	1132 ± 59	1000 ± 95	2308 ± 68
**L1156=**	3598T>C-3466T>C	TMD2	1299 ± 58	3298 ± 269	3167 ± 139	5429 ± 258	373 ± 24	1255 ± 5	1117 ± 96	2651 ± 138
	3600G>A-3468G>A	TMD2	1308 ± 55	3307 ± 201	3213 ± 160	5414 ± 261	373 ± 25	1299 ± 31	1151 ± 136	2695 ± 105
	3468G>A	TMD2	1312 ± 46	3232 ± 178	3186 ± 149	5514 ± 255	346 ± 16	1162 ± 21	1037 ± 77	2386 ± 60
**E60X**	178G>T	Lasso	207 ± 46	478 ± 136	393 ± 33	675 ± 93	79 ± 14	148 ± 29	130 ± 23	272 ± 48
**Q493X**	1477C>T	NBD1	198 ± 43	412 ± 73	384 ± 56	647 ± 97	69 ± 16	113 ± 17	107 ± 20	196 ± 34
**G542X**	1624G>T	NBD1	205 ± 40	459 ± 104	403 ± 43	652 ± 76	73 ± 19	117 ± 19	109 ± 15	205 ± 46
**R553X**	1657C>T	NBD1	203 ± 38	442 ± 77	402 ± 17	647 ± 80	69 ± 14	116 ± 21	106 ± 18	199 ± 33
**S912X**	2735C>A	TMD2	202 ± 49	428 ± 82	387 ± 35	664 ± 128	74 ± 16	125 ± 17	115 ± 21	225 ± 43
**W1089X**	3266G>A	TMD2	206 ± 46	448 ± 105	396 ± 51	690 ± 96	71 ± 16	124 ± 13	110 ± 14	198 ± 34
**Y1092X**	3276C>A	TMD2	210 ± 39	457 ± 112	402 ± 44	663 ± 67	70 ± 14	134 ± 14	118 ± 21	227 ± 22
	3276C>G	TMD2	210 ± 49	490 ± 132	418 ± 43	689 ± 124	74 ± 14	129 ± 21	124 ± 25	237 ± 37
**E1104X**	3310G>T	TMD2	195 ± 49	453 ± 159	390 ± 50	675 ± 118	68 ± 15	114 ± 12	104 ± 19	192 ± 31
**R1158X**	3472C>T	TMD2	220 ± 56	523 ± 135	447 ± 81	740 ± 110	86 ± 16	189 ± 11	152 ± 13	351 ± 26
**R1162X**	3484C>T	TMD2	206 ± 39	463 ± 108	402 ± 39	696 ± 90	73 ± 15	130 ± 15	129 ± 29	256 ± 10
**S1196X**	3587C>G	TMD2	962 ± 166	2220 ± 202	2055 ± 254	3143 ± 290	245 ± 8	856 ± 26	701 ± 95	1381 ± 57
**W1204X**	3611G>A	NBD2	971 ± 147	2170 ± 251	1897 ± 278	2866 ± 417	234 ± 5	806 ± 54	622 ± 75	1265 ± 32
	3612G>A	NBD2	916 ± 167	2002 ± 339	1736 ± 319	2472 ± 407	214 ± 9	690 ± 80	543 ± 75	1045 ± 33

Variant	Amino Acid Substitution	CFTR sub-domain	Parental Surface Immuno-staining Intensity (Apo)	Parental Surface Immuno-staining Intensity (VX-661)	Parental Surface Immuno-staining Intensity (VX-445)	Parental Surface Immuno-staining Intensity (VX-445 + VX-661)	CANX KO Surface Immuno-staining Intensity (Apo)	CANX KO Surface Immuno-staining Intensity (VX-661)	CANX KO Surface Immuno-staining Intensity (VX-445)	CANX KO Surface Immuno-staining Intensity (VX-445 + VX-661)
**W1282X**	3846G>A	NBD2	353 ± 29	908 ± 247	900 ± 149	1533 ± 295	108 ± 8	365 ± 3	332 ± 64	825 ± 146
**Q1313X**	3937C>T	NBD2	202 ± 52	461 ± 126	404 ± 50	668 ± 108	68 ± 16	113 ± 21	106 ± 22	187 ± 32
